# The assessment of job satisfaction for the healthcare providers in university clinics of Lubumbashi, Democratic Republic of Congo

**DOI:** 10.11604/pamj.2014.19.265.3138

**Published:** 2014-11-11

**Authors:** Tshamba Henri Mundongo, Yav Grevisse Ditend, Didier VanCaillie, Kaj Françoise Malonga

**Affiliations:** 1 Faculty of Medicine, School of Public Health, University of Lubumbashi, Lubumbashi, Democratic Republic of Congo; 2 Faculty of Economics and Management, University of Lubumbashi, Lubumbashi, Democratic Republic of Congo; 3 HEC-School of Management, University of Liege, Center for Research on Corporate Performance, Liege, Belgium

**Keywords:** Job satisfaction, healthcare providers, EFQM Model, Democratic Republic of Congo

## Abstract

**Introduction:**

In the world, the health policies are necessary to satisfy with efficiency the requirements of the quality management in the health sector. The laboratory of the academic clinics of Lubumbashi in Africa was inspired by the EFQM model to improve its performance and the quality of its services offered to the community. The aim of this survey is to evaluate the level of job satisfaction of the healthcare providers after implementation of the model.

**Methods:**

Qualitative study used an anonymous questionnaire consisted of 16 semi directional dichotomous and 12 according to four modality of the Likert's scale; to evaluate the job satisfaction of the healthcare providers. 40 workers are concerned and their informed consent is obtained. Epi Info 3.5.3 and SPSS 19.0 software, the Student t test and Chi-square test and the threshold set at p ≤ 0.05 were used. The mean score was calculated. Cronbach's ‘ coefficient and principal component analysis allowed the validity measurement of the questionnaire, and the correlations has been calculated.

**Results:**

This survey had a rate of answer of 80% on a set of all questionnaires. The Cronbach's coefficient of reliability is 0.72 on 40 complete observations with 12 questions. The Kaiser Meyer Olkin (0.564) and the Bartlett test is significant (χ^2^= 57, 30, p=0.001). The Physicians are very dissatisfied (2.363) against the nurses, and the biologists who are moderately dissatisfied (3 and 3.312). The relative results to the global satisfaction of the workers show a meaningful difference between the workers satisfied versus those non satisfied (p = 0.003). More of the half of the workers is satisfied after the setting up of the EFQM model.

**Conclusion:**

A certain number of the factors act together and simultaneously on the satisfaction of the workers particular in the health sector. The EFQM model permits the job satisfaction in the hospital because it combines several factors acting on the individuals.

## Introduction

The EFQM (European Foundation for Quality Management) model of excellence putsa particular importance on the assessment of the results of the quality management on the human resources. In the world, the health policies are adopted and adapted to answer the requirements of the quality management in the sector of health.

The laboratory of the academic clinics of Lubumbashi in Africa was inspired by this EFQM model to improve its performance and the quality of its services offered to the community.

The insurance quality imposed to the hospitals and the sector of laboratory in France to the security concerning blood transfusion and medicine is one example [1], the relative law for the backing the sanitary security of the products destined to the human is another example [2]; a legislation prolonged by an opposable regulation in the hospitals constituted by the good practices of transfusion exists in France [1, 2].

In the laboratory, the investigations of satisfaction of the physicians have already been achieved [1, 3, 4] but rarely those of the internal actors of the laboratory. In the city of Lubumbashi, rare are the hospitals that have a politics of assessment of the satisfaction of the human resources. Two major problems have urged the authority of these clinics to improve the performance of the medical laboratory units namely the cessation of financing by the partner CUD (University Commission for Development of Belgium) in 2007 obliging the customers to support themselves the laboratory fees and consequently, to push them searching for the best costs and services of quality.

An assessment of satisfaction of the hospital actors of the academic clinics of Lubumbashi is useful, because this establishment of healthcare does not possess the monopoly in the offer of the health care in a very competitive environment but must assure the cares of quality to answer the needs of health and for the fidelisation of his/her/its customers.

An investigation of this span is an interesting tool in the improvement of the quality as underlined by Chord Auger S. and al., [1]. Renewable in the time, it must also be discerned like a tool of follow-up of the committed corrective actions.

In a magazine of the literature on the topic, M. Beaumont [5] and J. Lérat-Pytlak [6] put respectively in evidence two “visions”, two “paradigms” of the management of the quality. A radical humanist perspective that while granting a primordial place to the man in the production and in the enterprise, driven to upset the distribution of information, the power and rewards. The management of the containing quality then the recommendation of a radical change within all enterprise, a cultural revolution [5] putting the accent on the importance of the teams within the organizations.

The team work is encouraged in the approach of the quality management. The incentive of the employees is based on the concept of empowerment, and a style of management based on the concept of “leadership”.

The number and the type of work teams in an organization constitute good indicators of the presence of a culture of the quality management, of the delegation of responsibilities and the level of involvement of the employees,is the fundamental question to know what motivates people to work, to push them to learn more and to develop new expertise? What incite them to participate and to imply themselves in the realization of the objective quality to improve the quality of their work?

The approach of the quality management treats the question of the incentive of the various manners. Besides the team work and other aspects of the incentive, the primordial aim is probably the importance given to the employees [6]; and their satisfaction opposite the accomplished work. This concept of“ empowerment or to can” expresses the confidence that one grants to the employees while delegating them some responsibilities, what can be a source of incentive but requires a good framing notably in the activities of global management.

It can take several shapes. It can result in a better sharing of information on the results of the enterprise. It also concerns the autonomy let to people to improve their work and their performances. It also milked for example to the latitude let to the employees of “first line” to act in the customer′s interest without having need to get a previous authorization. The idea to give the power to the employees is the basis of the school movement of the human relations.

The approach of the quality management is also compatible with other more recent theories of incentive to work, notably the theory of the features of the employment of Hackman and Oldham [7], the one of the need of realization of Mc Clelland (1961-1987) or the one of fixing the objectives developed initially by E. Locke [8].

The realization of the goals of any system passes by the incentive of people, and the most important incentive is the one that comes from people themselves. Deming gives out the hypothesis that people have needs of esteem and realization of oneself [9]; which conditions their incentives. And Herzberg F(1959) agrees to distinguish different factors of incentive [10], two sources of incentive, one intrinsic corresponds to innate qualities, the other extrinsic has the tendency to substitute them progressively to the first, or even to destroy it [11].

Deming thinks that the main source of the incentive is intrinsic to the individual, but the accent put on the extrinsic incentive has a destructive effect. The intrinsic incentive is one of the characteristic keys of the socially responsible behaviours [12] that means voluntary behaviors that benefit to the other («prosocial behavior»), without waiting external rewards and without giving up one necessarily [13]. For Shamir and al. [14], what motivates people intrinsically doesn′t only come from the idea to really make the things, or to feel competent or to exercise a power and a control on the things of “self esteem”.

It is a style of management that doesn′t consist in ordering and in controlling but to guide and to drag [15]. In this case, people are discerned worthy of confidence, experts in their work and capable to coordinate themselves in a voluntary manner.

According to the approach of the organizational development, the change will be a success if the administrators succeed in promoting values of involvement of the employees and a consensus, notably while improving the organizational life quality [16].

## Methods

The assessment of the level of satisfaction of the workers is important in the hospital context. In the EFQM model of excellence, the assessment of the level of satisfaction of the workers represents an obligation and a regular measure of efficiency at a time. It is established that the investigation allows knowing the efficiency of the reorganization setting up within the services [17].

The satisfaction in the workplace is an incentive support that allows the individual to exercise positively in the context of the organizations and to maintain an effort toward the organizational objectives; it is an internal psychological process. Reason why, the multi-dimensional and psychological approaches are used. It is not possible to motivate people directly, but only to create an environment auspicious to elevate their degrees of incentive [18]. It′s established that the incentive is a transactional process that depends on the adequacy between the individual and the organizational context in which one works and the broader societal context.14 workers or 70% of the laboratory staff have been interrogated and all answered to an anonymous questionnaire semi directional, fills individually after their free consent. This investigation of satisfaction has been achieved in two chronological times and a positive support from the Ethics Committee of the University of Lubumbashi has been obtained.

Confidentiality, the liberty to express his (her) opinions and his (her) observations has been guaranteed for every laboratory technician interviewee.

The questions have been proposed under “closed and coded” shape so that the staffs choose without difficulty the compatible answers with their opinions and their observations Another assertion “to specify” has been proposed, and permit to understand more on the studied phenomenon. The tool standardized by Peter Warr, Jhon Cook and Toby Wall (1979) has been used after adaptation to the environment of the academic clinics of Lubumbashi [19]. The encoding and the analysis of the data have been achieved while using the software SPSS 19.0 version 2010. The t test Student, the Chi^2^ test permitted the comparison of the mean score. The threshold of significance fixed (p < 0.05 and CI to 95%). The analysis of the validity and the reliability of the questionnaire have been made by the Cronbach′s α coefficient. The number of questionnaire measurement with an analysis of main components (ACP), the coefficient of interrelationship. The Bartlett test and the Kaiser-Meyer-Olkin measure of precision to the sample have been calculated.

## Results

This investigation had a rate of answer of 80% on a set of all questionnaires addressed to the health staff. The staff of the laboratory answered favorably (75%) to this investigating in comparison with the one of the other services (p <0.05). The Cronbach's α coefficient of reliability is 0.72 on 40 complete observations with 12 questions.

The analysis in main components (ACP) with an orthogonal rotation (varimax with normalization of Kaiser), the interrelationships of the answers between them and the interrelationships of the answers in every scale have been valued. The K.M.O (0.564) and the Bartlett test (Chi ^2^ =57.30, p = 0.001) and Anova test gives (Chi ^2^ = 7.877; p = 0.001) (Table 1)

**Table 1:** Table of the ANOVA test

	Sum of the squares	df	Average of the squares	F	P
Intergroup combination	6,084	2	3,042	7,877	0,001
Intra Class	14,291	37	0,386		
Total	20,375	39			

The results indicate that our investigated people reacted of varied manner to this investigation; 85% of the workers against 15% of chiefs of services. Their age is more than 31 years old for 90%, and 87.5% among them have an experience of more than 6 years (Table 2)

**Table 2:** Baseline characteristic of the hospital workers interviewed

Variables	Characteristic	Physicians	Nurses	Laboratory technicians	Chi^2^	p
Sex	Male	22,5%	15%	15%	5,806	0,055
	Female	5%	25%	17,5%		
Function, and	Chief of service	0%	10%	5%	4,660	0.097
	Personal performer	27,5%	30%	27,5%		
Age	≤ 30 years	5%	5%	0%	14,668	0,005
	31-45 years	22,5%	15%	25%		
	45 years +	0%	20%	7,5%		
professional experience	≤ 5 years	5%	5%	2,5%	5,385	0,250
	6-18 years	22,5%	25%	22,5%		
	18 years +	0%	10%	7,5%		

The results in the Table 3, indicates again that the physicians are the most unsatisfied of the group (2.363) against the male nurses and the biologists who declared more to be moderately unsatisfied (3 and 3.312).

**Table 3:** Distribution of the global satisfaction by professional category

Qualification of the staff	Mean Score	Std Dev
Nurses (n = 16)	3,312	0,704
Physicians(n = 11)	2,363	0,504
laboratory technicians(n = 13)	2,769	0,599

**Figure 1:**
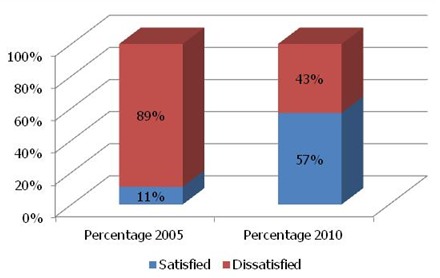
Temporary Assessment compared of the level of satisfaction for laboratory technicians

We compared the level of satisfaction in 2005 before the application of the EFQM model and after the quality management. The relative results to the global satisfaction of the workers show a meaningful difference between the workers satisfied versus those non satisfied (binomial Test = 0.050; p = 0.003). More of the 50% of the workers are satisfied after the implementation of the EFQM model (Figure 1)

**Figure 2:**
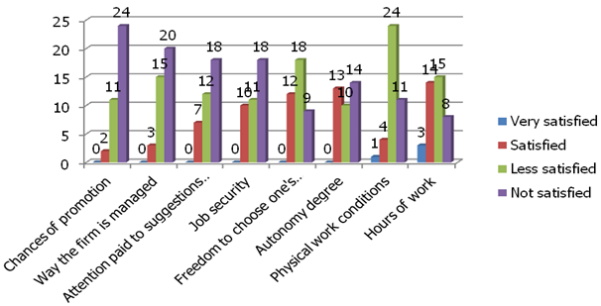
Results of variables model of Warr, Cook and Wall Scale used in the survey

Our results indicate that the workers are satisfied moderately for the variables“ attention granted to the suggestions, the fashion of security of the employment, the liberty of choice their work methods, the degree of autonomy and the work schedule”. The other variables indicate a strong dissatisfaction (Figure 2)

The remuneration is an important incentive factor. The results of our investigation, on this variable show that, (85%) workers of health are dissatisfied against (15%) which is satisfied for the whole remuneration.

**Figure 3:**
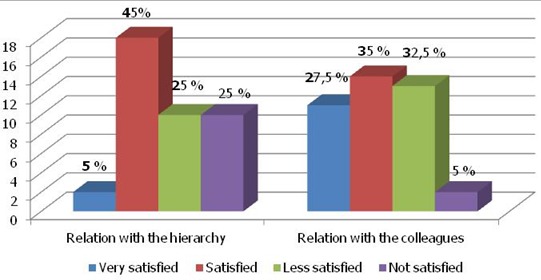
Workers satisfaction in the relation with hierarchy and colleagues

Concerning the quality of the relations within the organization, we note through the results that the relation with their colleagues is satisfactory for the set of the staffs (62.5%). The relation between the staffs and their hierarchies shows a satisfaction in the proportion of (50%) (Figure 3).

## Discussion

Some elements as the possibilities offered by the evolution of the career, the organizational support, the involvement to the decisions and the type of management produce positive effects on the salaried employee (implication, better quality of life?). But also on the organization (job satisfaction, better performances, decrease of the turnover or absenteeism) [20].

To understand the satisfaction of the workers is essential to understand the elements that sustain their strength of work. The job satisfaction, is defined as the effective response to one's job [8, 21, 22].

The questions of human resources management are the most important elements for the programs of the sector of health to improve the quality of the services. These are the engagement and the levels of incentive as well as the expertises of the humans that work in the organizations of the health sector and who have a meaningful impact on the performance, and to improve the best quality of the healthcare. The importance of better policies concerning human resources improve the performance of the health system is often put in evidence during these last decades [23, 24]. More and more challenges concern the human resources charged to provide the cares in the health systems financed by the public sector in the developing countries. Recent studies reported that low levels of job satisfaction among healthcare providers affect the patient-provider relationship and also compromise the quality of healthcare services [18].

The scheduling of the human resources doesn′t seem to be always a priority of the general politics of the health ministries in the development countries. The WHO, insist on the improvement of the capacities of human resources′ management for the improvement of the quality of the healthcare [25].

Our results indicate that the physicians are the most unsatisfied of the group against the nurses and the biologists who declared to be moderately unsatisfied. These results indicate not only the staff′s dissatisfaction but inform to sufficiency the global ambiance of the professional setting.

These observations push us to affirm the importance of really manage the human resources in the sector of health. While comparing the level of satisfaction between the year 2005 and 2010, we noted an improvement of (11% versus 57%) that we put in the account of the EFQM model(binomial Test = 0.050; p = 0.003).

To reinforce the capacities of the human resources in the programs of health and the physical work conditions for the human resources of health is important for the scheduling and the training of the human resources. A survey carried out by Freeborn (2001) reported that the workers of the health sector, who discern a big control, that their demands of work are reasonable and benefit in addition, they have the support of their colleagues, they have levels more raised of satisfaction and engagement towards the organization [26].

The relations between the workers are the reflections of the good general friendship that prevail in the organization and the manner whose casual association is formed inside an organization. The scores were (mean score 3.25 and 2.25) for the relation with the hierarchy and the colleagues, indicates a moderately dissatisfied of the relation with the hierarchy and a very dissatisfied of the relation with the colleagues. The results indicate that the relation between the staff is one of the elements to measure the satisfaction in the organization. These findings are confirmed by other studies that suggest that the relations with the colleagues are the best predictors of the satisfaction to work [27–30].

Among the major problems, we have the low wages and the staff′s incentive. The unequal and inequitable distribution of the work volume generates the staff′s bad performance and represents the main obstacle to the development of the health sector. The staff who works less than 30 hours per week has an incentive more raised in relation to those, who work of longer hours within the organizations [31, 32].

The new challenges are the frequent departures of the qualified staff toward the competitors and the more lucrative establishments that create a big insecurity for the organizations in the public sector. It is a sign of discontent of the human resources to work and an obstacle to the dispensation of better cares of health for the patients [22]. To understand the reasons of dissatisfaction to work is essential for all organizations. To discern the relation with the boss is of a primordial importance. The results of our investigation indicate a weak level of satisfaction (50%) for the relations with the hierarchy.

The studies have shown the constraints to work with a non cooperative boss. The interaction between superior and subordinate can entail a reduction of the satisfaction to work. The considerate and cooperative bosses are good facilitators of the groups for an increased productivity and for a better performance.

The substantial proofs attest the existence of a positive relation between the satisfaction to work and the relations between the direction and the workers [33, 34]. The remuneration is an important incentive factor. In this establishment, the results of our investigation on this variable show that (85%) workers of health are unsatisfied against (15%); who are satisfied for the whole remuneration.

The majority of the studies debate the importance of the financial incitements on the satisfaction. The financial incitement is considered like one main factor of retention of the human resources to the workplace.

The answer that the satisfaction to work is tributary of the incomes has been suggested in the studies by Clark and Oswald (1996). Fiji, Samoa, Tonga, Vanuatu, Papua New Guinea, Vietnam, Cambodia and Thailand, identified the low wages like a major reason of the weak incentive, dissatisfaction to working and the migration of the health workers [35–37]. There is on this day a debate of bottom on the efficiency to the performance bound to the remuneration in the development countries, for the setting of the health service in the public sector [38, 39].

To Cambodia, the financial incitements based on the performance of the health agents drove to better services of quality health with a growth of the productivity and a reduction of the expenses of use in the services [40]. But, the degree of autonomy that of healthcare providers possesses on their work is a very important variable to measure the intrinsic features of the employment [41].

The results indicate a weak level of satisfaction with regard to the autonomy and the incentive to work. The workers need more liberty to give satisfaction and to maintain their incentive.

The studies carried out by Murray show a positive association of the autonomy, the satisfaction to work and the incentive to work [42]; because the autonomy raises the satisfaction to work.

The employees will be motivated to use their responsibility of their work. The individuals′ age and their jobs offer more possibilities for the supplementary responsibility that is an intrinsic incentive, an important source of incentive. Some studies indicate that the responsabilization improves the level of interest of the human resources in their work [42].

In an ideal work environment, one can have the great opportunity to exploit his or her potential, drives to a bigger satisfaction to working and to raise a level of incentive. More studies also reinforce these findings [43].

The physical infrastructures are the fundamental condition for the working efficient of an organization. They have a positive impact on the satisfaction to the work of the healthcare providers. These findings join those (…) which declared that the bad physical work conditions in the public hospitals in India are a major reason of dissatisfaction of the staff [44]. The suggestions made by the staff in an organization can have a big impact on the performance of the institution. This variable also has an impact on the satisfaction of the employees to work [45].

The studies of Mulvany et al. (2002) indicate that the structures and the organizational processes affect the experience of the beneficiary of cares and the nature of the information that they use within the system of health. It′s established that the organizations that receive and treat the complaints of their agents efficiently have the more satisfied workers.

The recognition to working and the importance made to the suggestions of the healthcare providers are important factor of incentive for the workers. The recognition is bound directly to retention and the productivity [46, 47]. The studies also sustain a positive interrelationship between the quantity of recognition and the satisfaction of the workers.

According to Herzberg, the recognition of work is a motor of very important satisfaction. People to all levels of the organization want the recognition of their realizations on work and their successes must not be monumental to deserve recognition.

The opportunities of promotion have a positive interrelationship with the satisfaction to work. All things being otherwise equal, the possibilities of promotion have a positive interrelationship with the satisfaction to work. Herzberg in his theory to two factors insists on the fact that the possibilities of advancement in the rank are of strong incentives and drive therefore to the satisfaction to work. The weak satisfaction observed through our results would explain itself the modes of promotion that they judge not satisfactory. One study indicates that the promotion constitutes an aspect important of the mobility of the beneficiaries of healthcare [45].

The studies indicate a weak interrelationship between the possibility for the staffs to use their expertises and the satisfaction. However, some recent studies indicate that the security of employment is considered like a factor of satisfaction to work by a weak percentage of the salaried employees; which corroborates with the results of our investigation. For our results, the mean scores are weak between the three groups.

It can be assigned like an important factor for the weak level of satisfaction of the healthcare providers and the bad qualities of cares to the patients.

To the look of the results of this survey, one can affirm that the satisfaction to working is a complex phenomenon for which it is not easy to fix a factor determining the satisfaction or dissatisfaction to working [18, 48–50].

## Conclusion

This survey informs that a certain number of the factors act together and simultaneously on the satisfaction of the workers particular in the health sector. The dynamics of the relations between the factors is more important than a factor took in an isolated way. The EFQM model permits to evaluate the job satisfaction of the workers in the hospital because it combines several factors acting on the individuals.

The results show a meaningful difference in years 2005 and 2010 between the staffs satisfied versus those unsatisfied. More of the half of the workers is satisfied after the setting up of the EFQM model in the laboratory. The results will allow this service to improve the relation between the workers and their hierarchies, the autonomy, the remuneration and the consideration of the suggestions of the workers. These aspects that indicate dissatisfaction will deserve an attention to improve the incentive and the staff′s retention.

These results reinforce the interest for the organizations that produce services on the quality management to improve their performance.
